# The Value of Peer Learning in Undergraduate Nursing Education: A Systematic Review

**DOI:** 10.1155/2013/930901

**Published:** 2013-04-03

**Authors:** Robyn Stone, Simon Cooper, Robyn Cant

**Affiliations:** Monash University, School of Nursing and Midwifery, Berwick Campus, 100 Clyde Road, Berwick 3806, Australia

## Abstract

The study examined various methods of peer learning and their effectiveness in undergraduate nursing education. Using a specifically developed search strategy, healthcare databases were systematically searched for peer-reviewed articles, with studies involving peer learning and students in undergraduate general nursing courses (in both clinical and theoretical settings) being included. The studies were published in English between 2001 and 2010. Both study selection and quality analysis were undertaken independently by two researchers using published guidelines and data was thematically analyzed to answer the research questions. Eighteen studies comprising various research methods were included. The variety of terms used for peer learning and variations between study designs and assessment measures affected the reliability of the study. The outcome measures showing improvement in either an objective effect or subjective assessment were considered a positive result with sixteen studies demonstrating positive aspects to peer learning including increased confidence, competence, and a decrease in anxiety. We conclude that peer learning is a rapidly developing aspect of nursing education which has been shown to develop students' skills in communication, critical thinking, and self-confidence. Peer learning was shown to be as effective as the conventional classroom lecture method in teaching undergraduate nursing students.

## 1. Introduction

Nursing education studies have often focused on traditional teaching methods such as classroom lecture learning, a behaviourism-based teaching method based on passive learning [1]. More effective student-centric learning methods are now being utilized to encourage active student participation and creative thinking [2–4]. One of these methods is peer learning, in which peers learn from one another, involving active student participation and where the student takes responsibility for their learning. Despite being used for many years, one of the barriers to advancement of peer learning is a lack of consistency in its definition [5]. It is known by different interchangeable titles such as “cooperative learning,” “mentoring,” “peer review learning,” “peer coaching,” “peer mentoring,” “problem-based learning,” and “team learning.”

Peer learning has been used in education to address critical thinking, psychomotor skills, cognitive development, clinical skills, and academic gains [6–9]. One type of peer learning is problem-based learning (PBL) which is characterized by students learning from each other and from independently sourced information [10]. It is student centered, utilizing group work with the analysis of case studies as a means of learning. Alternatively “peer tutoring” involves individuals from similar settings helping others to learn, which may occur one-on-one or as small group sessions [11]. In nursing, high student numbers increase pressures [12] whilst varied and innovative teaching methods are beneficial [13] with peer learning offering a strategy that may be advantageous.

The Oxford Dictionary (2009) defines a “peer” as someone of the same age or someone who was attending the same university. The term “peer” can also refer to people who have equivalent skills or a commonality of experiences [14]. Both these definitions suit the concept of peer learning described here. The current study aimed to address the following research questions: (i) do undergraduate nursing students benefit from peer learning? and (ii) what approaches to peer learning are the most effective?

## 2. Methods

Operational terms as shown in Table 1 were developed after consulting several studies. Inclusion and exclusion criteria were defined. A search was conducted for peer-reviewed papers published in English between the years 2000 and 2010 that discussed any aspect of the curriculum for undergraduate or general nursing courses (e.g., clinical skills, communication, patient interaction, or theoretical knowledge).

The literature search was undertaken using the PICO algorithm of Participant, Intervention, Comparison, and Outcome and guidelines from The Cochrane Handbook [15]. For this study the following terms were used:

 participant—undergraduate nursing students,
 intervention—peer learning,
 comparison—classroom lecture learning,
 outcome—improvement in theory results or results of practical assessment or personal feelings relating to comfort, confidence, or competence.

A systematic literature search of multiple databases and search engines was undertaken using the keywords and the search strategy described in the following. The keywords used were student nurse, undergraduate nurse, peer learning, peer tutoring, peer mentoring, education, and opinion leaders. Also used were variations and truncations of these words, for example, peer education, nurse education, and problem-based learning. Each of the key words was searched for individually and then in combination with all others.

In addition, a number of key nursing journals such as Journal of Advanced Nursing and Journal of Nursing Education were hand-searched between the years 2000 and 2010. Snowballing, identifying suitable articles from the references of the selected studies, was then conducted to locate further studies.

Studies with all levels of evidence that met the criteria were included because studies in peer learning tended towards quasiexperimental, observational, or case study designs which are all lower on the hierarchy of evidence [16].

Two reviewers conducted a quality analysis of each study using quality criteria for qualitative and quantitative studies of the Critical Skills Appraisal Programme (CASP) [17]. They assessed methodology, validity, sample type, selection method, level of evidence, and any attrition rate and its effect (including biases), to determine selection. Consensus was reached through discussion. Data extraction and thematic analysis were undertaken to synthesize the data. Meta-analysis was not undertaken due to the inconsistent definition of “peer” and potential for bias if different methods of peer learning were combined. Although the primary review was conducted by a single researcher, inclusion and exclusion criteria were adhered to, using a transparent reporting process to allow the search to be reproduced.

Data were extracted and summarized in separate tables: quantitative studies (Table 2), qualitative studies (Table 3), and mixed method studies (Table 4). Drawing from this, data outcomes were collated into themes and subthemes; for example, the resources theme had three subthemes: faculty, students, and peers.

## 3. Results

**Figure 1:**
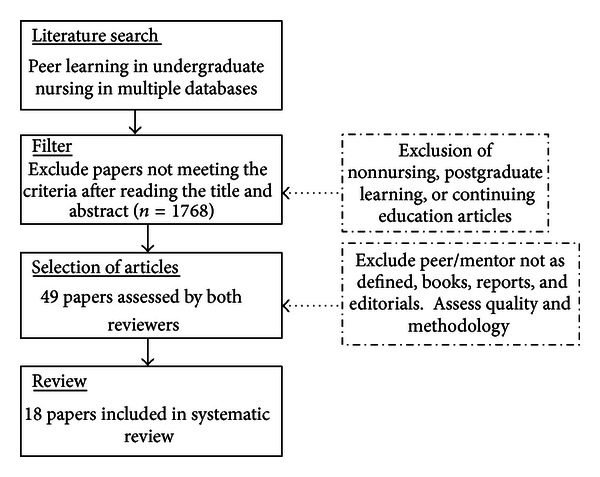
Flow chart of the systematic review selection process.

Initially, 1813 studies were screened using the aforementioned criteria and 18 studies were selected for review. A flow chart (Figure 1) shows the selection process including number of studies excluded at each stage and the reason for exclusion.

### 3.1. Characteristics of Studies

Participants were undergraduate nursing students from first to final year. In line with nursing demography the majority of participants were females. Participant numbers and study duration varied, for example, 15 students over a three-year period [18] and 365 students over a two-year period [7]. A variety of peer learning terms and methods were used. These terms included “peer mentoring” [19], “peer tutoring” [7, 20–23], “peer coaching” [24], “inquiry-based learning” [25, 26], “problem-based learning” [27–31], and “team learning” [32].

Of these studies, eight used a qualitative method, six utilized a quantitative method, and four used mixed methods (see Table 2). The quantitative studies favoured scaling and rating approaches (e.g., Likert scales), applying valid tools including the Californian Critical Thinking Disposition Inventory [28, 31], the Psychological Empowerment Scale [30], or the Nursing Ethical Discrimination Ability Scale [22] to collect data. The qualitative studies used a variety of collection methods such as participant observation [18], focus groups [19, 20, 25, 26, 33], individual interviews [23], and open ended short answer questions [27]. Combinations of these methods were used by the mixed method studies. In addition a variety of statistics software and applicable inferential statistics were used in the quantitative and mixed method studies, whilst the qualitative studies used applicable thematic approaches to analysis.

The analysis tools and tests used were presented (see Tables 1, 2, 3, 4, and 5) and considered as part of the quality analysis. Whilst quantitative studies give a definitive, measurable result, the use of qualitative studies in this paper examined how the participants felt about a different method of learning and how it impacted on them. These opinions would be important if peer learning was to gain acceptance from the students and peers. Qualitative studies had much smaller participant numbers and whilst larger numbers may have provided a more comprehensive sample, there were saturation and repetition of concepts even with the smaller samples.

Experimental studies with the highest level of evidence, that is, random controlled trials (RCTs), were not a common method of evaluating peer learning as they may not reproduce the true situation in an educational setting [34].

Eight studies used a comparison group [21, 22, 27–31, 35], and all except two [27, 31] were quantitative studies. Valid tools were used by 9 of the 10 quantitative or mixed method studies. It was concluded that valid tools measured what the authors intended, and hence data were an accurate representation of what had occurred and were credible. However, one study [21] failed to report the number of control and intervention group participants who passed their course. The qualitative studies documented the research process clearly, and the findings were consistent with the data provided. The details in description of the research process and findings varied between studies, whilst within individual studies, sample size, location, and the subjects taught were all potentially limiting; for example, small sample size, specific location, or subject may have caused a bias in the results, hence affecting the transferability of results [24, 31]. Publication bias was not evident as the included studies reported both the positive and negative results, for example, using comments from participants [26] or differences in means [35].

### 3.2. Effects of Peer Learning

Peer learning encouraged independent study, critical thinking, and problem solving skills. It could give students a sense of autonomy when they accepted responsibility for their own education. Peer learning was associated with increased levels of knowledge in a number of areas such as problem solving and communication [20, 25]. Tiwari et al. [31] showed that critical thinking was improved in students using PBL (*P* = 0.0048) whilst Daley et al. [20] reported that students showed improvement in cognitive and motor skills. An advantage of peer learning was that both groups learned and benefited from the interaction [23, 24]. The benefits differed between the students and peers with the peers gaining experience in communication and leadership, reinforcing their prior learning and discovering what they were capable of achieving in the mentoring/teaching fields [19, 24]. On the other hand, students gained confidence and experienced a decrease in anxiety when dealing with certain situations such as clinical placements. PBL was reported to be effective particularly in the theoretical learning component of education whilst peer tutoring, peer coaching, peer mentoring, and the use of role play as a form of peer learning were all effective, both in clinical and theoretical aspects of nursing education.

One negative aspect was related to anxiety levels. Group learning showed an increase in anxiety in both control and intervention groups [35], whilst other methods such as peer coaching showed a decrease in anxiety in the clinical setting [24]. The students indicated that having another person assist them decreased their anxiety levels which was pertinent when they were beginning their careers and were uncertain and anxious about what was expected of them [18]. Informal peer learning also benefited the students by providing them with “survival” skills that are not taught in lectures or text books, which in turn assisted in decreasing anxiety [18].

In addition to decreasing anxiety [24], many students showed an increase in satisfaction when peer learning was used [22, 29], appreciating having to think for themselves, problem-solve, and work as a team [20, 24, 27, 30]. The level of satisfaction is higher with peer learning [29] than the passive CLL method although some students still prefer CLL as it maybe better suited their individual learning style [22]. Whilst none of the studies in this paper directly investigated the link between course satisfaction and academic results, Higgins [21] noted a decrease in attrition rate but did not specify student satisfaction levels.

Four studies [20, 23–25] examined confidence in students when utilizing peer learning. They showed a subjective increase in confidence levels when completing clinical skills, problem solving, and critical thinking. In addition, 11 studies indicated that students appreciated interactive learning sessions and the emphasis on active participation, which encouraged them to take ownership and responsibility for their own learning.

### 3.3. Utilization

Peer learning was utilized in multiple situations from teaching ethics [22] and critical thinking [31] to helping students deal with emotional situations [19] with patients. In clinical situations peer learning was successful and improved integration into the ward situations [18] and students' confidence when dealing with actual patients rather than a simulator [20]. Further, a number of studies identified outcome measures showing an improvement in either an objective or subjective assessment.

## 4. Discussion

The purpose of this research was to ascertain whether undergraduate nursing students benefit from peer learning. Sixteen of eighteen studies demonstrated positive aspects to peer learning with outcome measures showing improvement in either an objective effect or a subjective assessment (such as a self-rated increase in student confidence). Furthermore, learning from peers was shown to be acceptable to most students. Much of the research into peer learning concentrated on formal peer learning with the evidence supporting the concept that peer learning may be an equally, if not more, effective method of delivering information in undergraduate nursing education.

Peer learning can be utilized to pass information to large groups of students with less faculty member involvement. At a time when there is pressure to train more nurses and minimize costs [12], peer learning could utilize resources more effectively with students teaching and supervising more junior students, thus decreasing the demand on the responsible faculty members. Therefore it may have cost benefits for managing some aspects of nursing education; however this theory requires further investigation as it was not the focus of any of the papers mentioned in this paper.

Regardless of any decrease in active involvement by lecturers, the need for student supervision remains important. If peers are not knowledgeable or do not have the appropriate skills, then they cannot accurately pass information onto another student. The learning of inaccurate information could potentially cause issues for students when these inaccuracies were demonstrated in exams and on clinical placements. Without supervision, learning may not be effective as shown in an earlier study by Parkin [4] who found that observation and supervision were required in all peer learning to ensure that correct and current information was being exchanged.

### 4.1. Advantages and Disadvantages

Peer learning may result in information being more readily accepted by a student as individuals often turn to others who have similar experiences, for advice and guidance. This could decrease anxiety associated with learning due to familiarity of the peer with the student's issues. As noted in the results, anxiety may occur when individuals are exposed to new concepts, whether they are novice or proficient learners [36]. In prior research [37], peer learning helped to decrease the student's anxiety and assisted them to fit into a ward situation and feel like part of the team. This sense of belonging has social implications particularly when it is known that learning occurs more effectively when there is socialization [38, 39].

Further, social interaction and collaboration between peer and student may have contributed to an increased learning curve and acquisition of further knowledge than would have occurred if students were studying independently. This was illustrated in this paper with students who had been in danger of failing and had received peer tutoring [21]. They gained additional knowledge and improved their academic result. This method may also allow junior students to problem-solve issues with their patients more independently and care for higher acuity patients, leading to an increase in their self-confidence. This concept was previously reported by Vygotsky [40], Aston and Molassiotis [41], and Secomb [5] who found that peer learning promoted self-confidence in junior students whilst assisting senior students with mentoring and teaching skills. Secomb [5] also showed that peer learning was an effective learning tool in clinical situations with both nursing and other health professionals. Peers perceived an increase in patient care competence when peer learning was utilized. Secomb [5] noted, however, that issues such as inappropriate pairing of students and peers should be addressed prior to the intervention.

Student satisfaction may play a part in scholastic achievement through acceptance of an active learning method such as peer learning. Four studies investigating this component [22, 28–30] discovered that students were satisfied with peer learning as the educational method. This may be because the student takes more responsibility and actively participates in their education, giving them a sense of autonomy. Whilst Ozturk et al. [28] reported a higher satisfaction level with PBL but no significant difference in academic scores, previous studies have shown a positive association between student satisfaction and grades achieved [42, 43].

Peer learning may also be more successful when peers are close in experience or stage of training as it provides a more relaxed, less intimidating, more “user friendly” learning experience than sessions conducted by registered nurses. Prior to the current study, El Ansari and Oskrochi [44], Eisen [45], and Secomb [5] also reported this finding whilst more recently Bensfield et al. [46] reported that first year students had comfortable learning with more experienced peers.

There is, however, a different perspective. Whilst peer learning has been used for years and might be becoming the norm, students may not be fully familiar with it and therefore be apprehensive about what it offers. Some students reported anxiety and apprehension when taking part in peer learning which was linked to feeling responsible for another's education [25, 26], being underprepared [23] or concerned that their own grades would be negatively affected by group work or dynamics [25, 26, 32]. Further, it was reported that enforcing the educational role of a peer may lead to resentment, particularly if the nurse felt unprepared or unwilling to undertake the role [26]. Previously Bensfield et al. [46] showed that whilst nurses have a responsibility to teach others, many are reluctant to do so as they feel unprepared for the role. Due to these issues, students who are familiar and comfortable with CLL may continue to prefer this learning method over peer learning.

### 4.2. Confusing Terminology Used in Peer Learning

Finally, this paper raises issues around the confusing terminology used to refer to peer learning. As mentioned, multiple terms such as peer teaching, peer mentoring, and peer coaching were used interchangeably with debate about whether they meant the same thing or referred to subtle differences in meaning. A clearer definition of each of the terms is needed to increase the rigor of nursing education research, as was also raised by Secomb [5], Eisen [45], and McKenna and French [47] who found a lack of clarity as to what peer education entailed due to the interchanging of terminology. We suggest amalgamation of some of the terms, for example, team learning and cooperative learning or peer mentoring and peer coaching and having distinct differences in definitions between other terms.

## 5. Limitations

Some limitations of this study were recognized. Only studies published in English were included. Despite a rigorous search strategy, some relevant articles may have been omitted owing to the search terms used. The mix of study designs and various methods of reporting meant that direct comparison between studies was limited. Some studies were location- or topic-specific, and some studies collected data using indirect outcome measures. However, the collection and review of the diverse papers that were selected offered the best option for determining the overall impact of peer learning in education of nursing students.

## 6. Conclusion

Peer learning: learning from others who possess a similar level of knowledge, is becoming a part of nursing education. This study showed that undergraduate nursing students could benefit from peer learning, with an increase in confidence and competence and a decrease in anxiety. Their peers also gained skills to prepare them for their role as a registered nurse. Conduct of peer learning within the curriculum was shown to require adequate academic supervision to be effective. It was difficult to ascertain the most effective learning methods because of the inherent variation between study methodologies, terminology, subjects, and settings. Inconsistency terminology was identified as a problem that should be addressed in order to provide clarity in future research.

## 7. Further Research

Further research is needed to fully investigate peer learning with the use of larger samples, various targeted curricula, courses, and locations to increase the validity of studies. The cost effectiveness of peer learning should be further investigated and compared to that of CLL to ascertain how this option could impact nursing education in terms of resources, time management, and effectiveness.

## Figures and Tables

**Table 1:** Operational definitions.

Term	Definition
Peer	A person with a comparable or slightly higher level of knowledge and experience to the learner. A person of any age can be a peer, that is, a third year student tutoring a first year student but the peer must be an undergraduate nursing student.
Peer learning	Gaining, refining, or improving knowledge from interaction with a peer as defined previously.
Mentor	A peer, who supports, guides, and educates a learner.
Undergraduate nursing student	A nursing student at any stage of study prior to registration.

Sources: [10, 14, 45, 48–51].

**Table 2:** Summary of included studies.

Author	Design	Intervention and comparison	Country and setting
Broscious and Saunders (2001) [24]	Mixed method Group interview and questionnaire	Peer coaching	Junior and senior year students: Christopher Newport University in USA
Christiansen and Bell (2010) [19]	Qualitative Interpretive	Peer learning	UK University preregistration nursing course
Christiansen and Jensen (2008) [33]	Qualitative Ethnographic	Role playing	Third year student nurses in Norway
Cooke and Moyle (2002) [27]	Qualitative—case studies	PBL versus CLL	Second year students at university in Australia
Daley et al. (2008) [20]	Qualitative Observation, focus groups, and journaling	Peer tutoring	Ohio State University, College of Nursing Students
Feingold et al. (2008) [32]	Mixed methods Observation and interviews	Team learning	First year student nurses: university in South Western USA
Goldsmith et al. (2006) [7]	Mixed method Portfolios, clinical assessment, and questionnaires	Peer learning	First/third year students: Clinical Practice Unit (CPU) of a nursing school in Australia
Higgins (2004) [21]	Quantitative Quasiexperimental	Peer tutoring versus nonpeer tutored	Medical-surgical undergraduate nursing course: Texas, USA
Hughes et al. (2003) [35]	Quantitative Pretest/posttest crossover	Peer group experience versus nonpeer group experience	Baccalaureate school of nursing in USA
Horne et al. (2007) [25]	Qualitative Focus group interviews and use of fourth generation evaluation	Enquiry-based learning	Second year students at a higher education setting in the UK
Lin et al. (2010) [22]	Quantitative Experimental	Peer tutoring. PBL method in class versus CLL	Nursing ethics classes: university in Taiwan
Loke and Chow (2007) [23]	Qualitative Focus groups and individual interviews	Cooperative learning	Second/third year student nurses: university in Hong Kong
Morris and Turnbull (2004) [26]	Qualitative evaluation Focus group interviews, field notes	Peer tutoring	3-year undergraduate nursing course in the UK
Ozturk et al. (2008) [28]	Qualitative Descriptive, analytical	PBL versus CLL	Fourth year undergraduate student nurses from two universities in Izmir, Turkey
Rideout et al. (2002) [29]	Quantitative survey Cross-sectional analytical design	PBL versus CLL	Baccalaureate school of nursing and CLL programme in Canada
Roberts (2008) [18]	Qualitative Interpretive ethnographic	Informal peer learning	Undergraduate students over a three-year course in a range of acute settings including intensive care, general surgical, rehabilitation, and medical wards in the UK
Siu et al. (2005) [30]	Quantitative Descriptive, correlation survey design	PBL versus CLL	Two universities in Ontario, Canada
Tiwari et al. (2006) [31]	Mixed methods Random controlled trial and survey	PBL versus CLL	First year student nurses: university in Hong Kong

PBL: problem-based learning, CLL: classroom lecture learning.

**Table 3:** Quantitative studies.

Author/year	Aims	Sample/method	Power calculation/software/analysis tool used	Outcomes	Generalization and confounding factors	Further research
Higgins (2004) [21]	To determine relationship between academic performance, retention, and participation in a peer-tutoring programme.	Purposive sample: 26 at-risk students were offered peer tutoring *n* = 20 peer tutored; *n* = 6 in nonpeer tutored group	(i) Power calculation not given. (ii) Fisher's exact test; a two by two bivariate frequency distribution.	Significant relationship between academic performance/retention and participation in a peer-tutoring programme. Attrition rate decreased from 12% to 3%.	Difficult to generalize—no randomization; those who sought peer tutoring may be more motivated to pass. Small sample limited the finding.	Other nursing programs should duplicate peer tutoring for at-risk students in their courses

Hughes et al. (2003) [35]	To investigate effect of an informal peer group experience on baccalaureate nursing students.	Convenience sampling Voluntary selection *N* = 128	Insufficient power to detect group differences; multiple tools assessed, anxiety, depression, professional socialization, and general self-efficacy.	No significant differences between groups, but increased depression (mean 0.83, SD 0.55) and anxiety (mean 2.40, SD 0.81).	Restricted to two universities. Not generalized. Anecdotal accounts: programme was beneficial maintaining contact with others from group, assisting coping skills.	Need for longitudinal study on nursing at varying stages in course

Lin et al. (2010) [22]	To compare the educational results of peer tutor problem-based learning and conventional teaching in nursing ethics education.	Convenience sampling Randomly assigned to control and intervention groups control = 70, intervention = 72	No power calculation done. Nursing Ethical Discrimination Ability Scale; Learning Satisfaction Survey. Internal consistency reached 0.8 and split-halves reliability was 0.76.	Both methods effective for teaching ethics with PBL (*P* < 0.01) slightly more effective than conventional lecture (*P* = 0.020).	Only one university. Limited training of peers, no evaluation of how peers did their job. Teaching materials/teachers may have influenced result. Students used to CLL may prefer this.	Method needs further validation.

Ozturk et al. (2008) [28]	To compare effects of PBL and traditional education on senior undergraduate nursing students' critical thinking dispositions.	Convenience sampling: PBL = 52, CLL = 95	Power calculation not given. California Critical Thinking Disposition Inventory. Independent *t*-tests for between-group differences.	PBL group showed higher levels of critical thinking than control group (*P* < 0.01); both groups in medium range for critical thinking (between 240 and 300 on CCTDI).	Two universities, voluntary participation, but good response rates. Generalization could be made with reservations as corroborate previous studies.	Alluded to development of a tool to measure critical thinking that is more specific to nursing.

Rideout et al. (2002) [29]	To compare graduate baccalaureate students in a PBL curriculum with others in a CLL programme.	Convenience sampling: PBL = 75, CLL = 52	No power calculation given. *P* < 0.01. *t*-tests, ANOVA for statistical analysis.	No differences in final exam results (*P* = 0.21) Passed: PBL 93% and CLL 98%)	Restricted to two universities.	Future studies to be longitudinal in design and relying less on self-report measures.

Siu et al. (2005) [30]	To evaluate if nursing students enrolled in PBL programme had higher perceptions of empowerment than those in conventional learning lecture (CLL).	Convenience sampling: PBL = 83, CLL = 70	No power calculation given. Conditions for Learning Effectiveness Questionnaire (CLEQ). Psychological Empowerment Scale, Teaching-Learning Strategies Questionnaire, Clinical Problem-Solving Scale. ANCOVA; Spearman's Rho applied.	PBL experienced greater structural empowerment (*P* = 0.001). Students taking responsibility for their own learning helped develop effective communication, listening, problem solving, and collaboration skills.	Sample from only two universities. Self-selection and self-reporting questionnaires were a potential problem.	Need for longitudinal investigation and replication with samples from several nursing programs.

**Table 4:** Qualitative studies.

Author/year	Aims	Participant selection	Assessment measures	Outcomes	Suggested research
Christiansen and Bell (2010) [19]	To explore impact of peer learning initiative to facilitate mutually supportive learning between student nurses.	Purposively selected. *N* = 54.	Focus group interviews. Narrative date analyzed thematically. Data collected over 18 months with 3 cohorts of students.	Active support from peers can reduce negative feelings experienced by first year student nurses. It assists coping and decreases attrition rates. Peers benefited by gaining confidence and increased readiness for registered practice.	Explore impact of peer learning on mentors in the workplace.

Christiansen and Jensen (2008) [33]	To explore classroom-based peer learning enabling students to cultivate their modes of expression.	Voluntary selection from 288 students doing third year communication course; 16 students took part in role play and 8 in focus group interview.	Observations and focus groups.	Provide tools that can be used in clinical practice. Students learnt from each other and appreciated being able to experience different roles.	Further study on students emotional learning during periods of clinical practice.

Cooke and Moyle (2002) [27]	To explore and evaluate the use of PBL over a one-month period in programme that uses CLL.	130 students—100 responded to questionnaire.	Open ended questionnaire.	Gave more control over learning and added responsibility, promoting independent, self-directed learning. PBL was enjoyable.	None mentioned.

Daley et al., (2008) [20]	To evaluate a peer-tutoring approach to teaching designed to meet needs of two different levels of students.	All first year and some senior students in a leadership and management course.	Observations, conferences, focus groups, and journals. Multiple levels of evaluation.	Assignment of higher acuity patients to first years. Confidence of weaker students increased. Better understanding of role of team member, improvement in cognitive and motor skills, increased comfort in asking questions/discussing concepts, and awareness of a future role as mentor.	Additional work and formal evaluation of programme.

Horne et al. (2007) [25]	To evaluate student and facilitator perspectives of hybrid model of problem-based learning.	All 121 second year students and 15 facilitators.	Focus group interviews and use of Fourth Generation Evaluation (nominal group technique).	PBL aided development of independent learning skills, self-awareness, and confidence skills. Improved interpersonal/communication skills but depended on group collaboration and concerns about teamwork and group dynamics.	Further study of effectiveness for large groups of students >100.

Loke and Chow (2007) [23]	To facilitate the development of “cooperative learning” among nursing students through peer tutoring.	Voluntary selection of 14 third year students as tutors and 16 second year-students as tutees.	Focus groups and individual interviews at half way and end of 10-week tutoring period.	Positives included enhancement of deep learning, critical thinking, problem solving, communication skills, confidence in own learning, and time management. Negatives included participants being late or feeling of not having adequate knowledge to share with one another and mismatched personalities.	Further studies to examine peer-tutoring effects on students in all years.

Morris and Turnbull (2004) [26]	To explore viability and value of using student nurses as teachers in inquiry-based nursing curriculum.	A purposive sample of 240 student nurses (36%) participated in interviews.	Focus group interviews, field notes, interviews audio-taped, transcribed, and analyzed using thematic analysis.	Student nurses were uncomfortable with this method and thought it mostly inappropriate, although it may be appropriate in some circumstances. Use of parallel resource sessions in a nonfacilitated forum is problematic. Participants did not think that skills were transferable.	Further research required on the use of students as teachers.

Roberts (2008) [18]	To explore if students learn from each other and how, when/where this takes place.	Voluntary selection of 15 students.	Audio-taped conversations of students and field notes; thematic analysis.	Friendships are important to learning. Peer learning in clinical practice is informal and considered part of the job. It helped students to ask questions and contribute to the ward. Students were approachable and had more time for teaching. Students valued peers in clinical setting helping each other to cope with demands of clinical practice.	Replicate study with other groups of students in different locations and branches of nursing.

**Table 5:** Mixed method studies.

Year/author/	Aims	Setting	Participant selection	Assessment measures	Outcomes	Generalizations and limitations	Suggested research
Broscious and Saunders (2001) [24]	To implement a strategy to reduce student anxiety during their first clinical experience.	Junior/senior year students in university in USA	20 senior students and 25 junior students. Convenience sample of all students in year.	Group interview and Likert scale survey: scale had statements presented as description of student experiences.	On 5-point Likert scale (only means reported) students reported decreased sense of anxiety (mean 4.29), increasing confidence (mean 4.08), improved organization (mean 3.91), and perception of being a member of the team (mean 3.83). Senior students practiced leadership skill (mean 4.53), established priorities (mean 4.4), and developed plan of care (4.4). Reiterated amount learnt by senior. Impact of coach decreased in subsequent clinical session.	Small sample and voluntary selection limits generalization.	Further study by modifying and different sites and larger sample.

Feingold et al. (2008) [32]	To evaluate student perception of team learning and effect on class interaction and engagement.	First year student nurses at a university in south-western USA	First year students in course. Convenience sample: for interview—10%, responses—21%.	Observation and interviews (Qualitative). STROBE classroom observation tool (Quantitative).	Team learning promoted student engagement and was predominantly interactive. Over 305 minutes, most students were engaged/on task for 84% of time, with 70% of time being learner to learner engagement and further 8% of time being used for self-engagement. Main behaviour was learner to learner engagement. Recognized benefit of discussion of problems.	The concept could be generalized. Small sample may not be representative of first year students (nonrandom, self-selected).	Further study to help refine team learning process and to guide implementation of interactive learning strategies.

Goldsmith et al. (2006) [7]	To explore impact of peer learning on anxiety of first year student nurses when undertaking clinical skills assessments.	First/3rd year students in Clinical Practice Unit (CPU) in Australia (Conducted over two years and two campuses)	First/3rd year students in 2-year time frame. First year cohort of 187; 3rd year: 178.	Portfolio submission as 3rd year, clinical skills assessment for first year students. Both groups completed questionnaires.	Learning enhanced by experience. Evaluation completed after 2 years; good representations of both campuses/years. Learning enhanced by experience. Positive answers to questions were given by a majority of both 1st/3rd years with 3rd years more likely to give positive responses.	Only two campuses of one university. Late development of assessment tool with not all students given opportunity to complete questionnaire. Voluntary completion of questionnaire which may not have been representative.	Not reported.

Tiwari et al. (2006) [31]	To compare effects of problem-based learning and lecturing approaches on development of students' critical thinking.	First year student nurses in one university in Hong Kong	All 79 students in first year randomly assigned to control and intervention groups. PBL = 40, CLL = 39. Selection by ballot.	Multivariate regression, 1 and 2 sample* t*-tests.	No significant difference between groups at initial testing (*P* = 0.48). 2nd and 3rd data collection points showed significant difference in overall CCDTI scores between PBL and control groups (*P* = 0.0048, *P* = 0.0083). The 4th collection point showed higher score on the CCDTI for PBL group but nonsignificant difference (*P* = 0.1159). PBL students found their course more enjoyable/inspiring than lecture group.	Only one university and a small sample. Results may have been influenced by how lectures were conducted. Use of self-reporting tool.	Monitoring of groups critical thinking in subsequent years.
